# Glycolytic Reprogramming in Endometriosis: Biological Basis and Emerging Targets for Disease-Modifying Therapy

**DOI:** 10.3390/jcm15155774

**Published:** 2026-07-23

**Authors:** Catarina Sobral, Julieta Afonso, Jorge Correia-Pinto, Fátima Baltazar, Cristina Nogueira-Silva

**Affiliations:** 1 Life and Health Sciences Research Institute (ICVS), School of Medicine, University of Minho, 4710-057 Braga, Portugal; catarinasobral@med.uminho.pt (C.S.);; 2 ICVS/3B’s—PT Government Associate Laboratory, 4710-057 Braga/Guimarães, Portugal; 3 Department of Obstetrics and Gynecology, Braga Local Health Unit, 4710-243 Braga, Portugal; 4 Department of Pediatric Surgery, Braga Local Health Unit, 4710-243 Braga, Portugal

**Keywords:** endometriosis, therapeutics, glucose metabolism, cancer, repurposing

## Abstract

Endometriosis is a chronic gynecological disease affecting approximately 10% of women of reproductive age and up to 40% of women with infertility, with a significant impact on quality of life due to pain and reproductive impairment. Its etiology remains unclear, although several mechanisms have been proposed, including retrograde menstruation and immune dysfunction. Increasing evidence highlights similarities between endometriosis and cancer, particularly regarding selected hallmarks such as sustained cell proliferation, angiogenesis, inflammation, invasion, and immune dysregulation. Notably, alterations in glucose metabolism have been identified, suggesting a metabolic reprogramming resembling the Warburg effect in cancer. This review examines clinical aspects, therapeutic challenges, and emerging evidence for Warburg-like glycolytic metabolism in endometriotic lesions, which favors aerobic glycolysis over oxidative phosphorylation to evade apoptosis and promote survival in hypoxic microenvironments. These cancer-like hallmarks—shared with malignancies—suggest repurposing glycolytic inhibitors as targeted therapies to disrupt disease progression beyond symptom palliation. However, most evidence remains preclinical, and important challenges regarding disease heterogeneity, target validation, and safety still need to be addressed.

## 1. Introduction

Endometriosis, defined by the presence of endometrial glands and stroma (connective tissue) outside the uterine cavity, is an estrogen-dependent gynecological disorder that primarily affects women of reproductive age. It is a complex disease characterized by a variety of phenotypes (anatomically and clinically) [1,2].

It is impossible to identify a single source that justifies the etiopathogenesis of endometriosis. Multiple theories have emerged over the years that have contributed to a better understanding of the disease’s onset, Sampson’s theory of retrograde menstruation from 1927 being the most widely accepted [1]. According to this theory, during menses, eutopic endometrial cells flow backward from the uterine cavity through the fallopian tubes into the peritoneal cavity, where they may give rise to endometriotic implants, thereby causing chronic inflammation [3]. Moreover, it has been shown that the incidence of endometriosis is increased in women with genital tract obstructions (uterine septum, cervical stenosis, and imperforate hymen) because of increased tubal reflux [4,5]. Although attractive, it is easily understandable that this theory does not exhaust the explanation for the occurrence of this disease, since up to 90% of women have retrograde menstruation and the majority do not develop endometriosis [6].

Recently, numerous factors, including immune responses, inflammation, aberrant endocrine signaling, genetics, and other processes related to cell survival, proliferation, and metabolism, have been associated with the development of endometriosis [7]. In the following sections, we will review the clinical aspects of the disease, its current management options, and the associated challenges. Among the emerging disease-modifying mechanisms under investigation, metabolic reprogramming has attracted growing attention for its potential role in lesion survival, persistence, and progression. Most available studies investigating glycolytic reprogramming have been performed in selected lesion types, particularly ovarian endometriomas and stromal cell cultures. Whether metabolic alterations are equally present across superficial peritoneal lesions, deep infiltrating endometriosis, and ovarian disease remains incompletely understood.

This narrative review was based on literature retrieved from PubMed up to February 2026 using combinations of the terms “endometriosis”, “glycolysis”, “metabolism”, “Warburg effect”, “GLUT”, “LDHA”, “PDK”, “MCT”, and “metabolic therapy”. Relevant original studies, reviews, and preclinical investigations were included according to their relevance to the topic.

## 2. Clinical Aspects of Endometriosis

**Figure 1:**
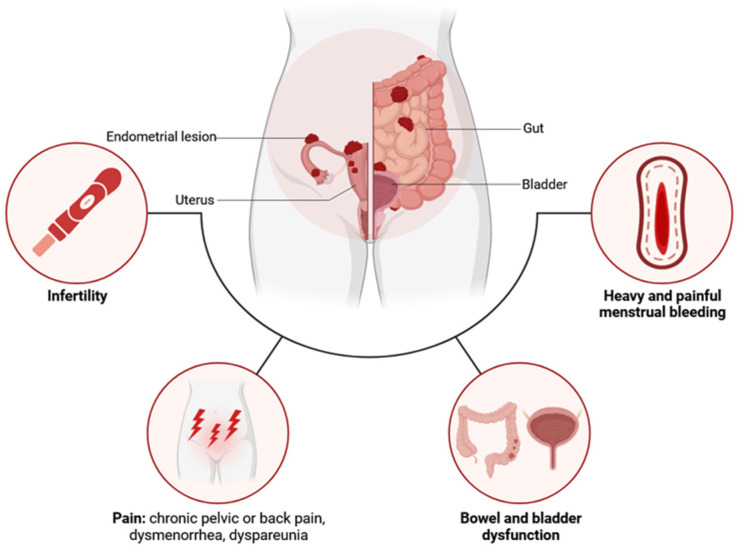
Clinical manifestations and systemic impact of endometriosis. Endometriosis is associated with a broad spectrum of symptoms, including chronic pelvic pain, dysmenorrhea, dyspareunia, dyschezia, dysuria, infertility, fatigue, and psychological distress. Symptom severity may vary considerably among patients and does not always correlate with lesion extent. Original figure created by the authors using BioRender.

Endometriosis represents a significant burden to women of reproductive age and is associated with high morbidity [8,9]. As in the uterus during the menstrual phase, endometrial glands and stroma outside the uterine cavity desquamate, leading to chronic pelvic pain, dysmenorrhea, dyspareunia, and heavy menstrual bleeding (Figure 1). Women may also report bowel and bladder dysfunction, abnormal uterine bleeding, and low back pain [10,11,12]. Importantly, beyond physical symptoms, endometriosis has a significant negative impact on quality of life, sexual satisfaction, fertility, and couple relationships, contributing to increased psychological morbidity in both patients and their partners [13].

Typically, pelvic endometriosis includes superficial peritoneal lesions, deep endometriosis, and ovarian endometriomas [14,15,16]. Occasionally, there may be endometriomas reported on the anterior abdominal wall, umbilicus, breast, hymen, pancreas, liver, gallbladder, kidney, urethra, vertebrae, bone, peripheral nerves, spleen, diaphragm, central nervous system, lung, etc. [17,18,19,20,21].

There is still a lack of correlation between the severity of endometriosis (in imaging and laparoscopy), the duration of the disease, and symptoms [22]. Regarding severity, the factors that lead to the progression of endometriosis, including the transition from minimal/mild to moderate/severe stages, are not fully understood [23,24]. Furthermore, there are different possible locations for endometriosis implants, and it is not known what determines the location in each woman or the predictable symptoms present in each of them. It is still unknown which women are at increased risk of extra-pelvic site occurrence [25,26].

### 2.1. Management of Endometriosis

Given its challenging management, endometriosis should be looked at as a chronic disease, requiring lifelong treatment with limited results, as shown in daily practice. Current management is therefore largely focused on symptom control and limiting disease progression rather than achieving a definitive cure, reflecting the still incompletely understood etiology and the chronic nature of endometriosis [27].

#### 2.1.1. Pharmacological Management

Endometriosis patients may be offered nonsteroidal anti-inflammatory drugs (NSAIDs), estrogen–progestin and progestin contraceptives, gonadotropin-releasing hormone (GnRH) analogues (agonists and antagonists), and aromatase inhibitors. However, there is no evidence for choosing one treatment over another, so treatment is selected based on a patient-shared decision, side effects, and contraceptive needs [28].

Regarding NSAIDs, there is no high-quality evidence demonstrating the efficacy or superiority of these drugs compared to others [29]. They should be supplemented with hormonal therapy, unless fertility wants to be preserved. Moreover, selective cyclooxygenase (COX) 2 inhibitors should be avoided if women are trying to conceive, as it has been found that these drugs may impair ovulation [30,31,32].

Hormonal therapy is a cornerstone in the management of endometriosis, primarily aiming to suppress ovarian activity and induce a hypoestrogenic environment, thereby reducing the proliferation and activity of endometriotic lesions. Combined estrogen–progestin contraceptives are widely used and can be administered continuously to improve symptom control, particularly pain. However, progestin-only therapies represent an equally important first-line option, but with no clear evidence supporting the superiority of combined formulations over progestins alone. Among these, dienogest has shown particular efficacy in reducing endometriosis-associated pain and lesion size, with a favorable tolerability profile, making it a well-established option for long-term treatment [33,34].

In many cases, women will continue to experience symptoms, and GnRH agonists are used to decrease pituitary secretion due to receptor downregulation and desensitization, leading to suppression of ovarian estrogen production. Due to their side effect profile, treatment duration is typically limited to 6 months [35,36]. GnRH antagonists, in contrast, induce immediate pituitary suppression and a dose-dependent hypoestrogenic state, while offering greater flexibility in dosing compared to GnRH agonists [37].

Regarding aromatase inhibitors (AIs), they are used in women with severe and refractory pain as third-line therapy. In premenopausal women, AIs are usually combined with progestins, estrogen–progestin contraceptives, or GnRH agonists to suppress folliculogenesis and prevent ovarian stimulation, since AIs alone can lead to ovarian follicular cyst development [38].

#### 2.1.2. Surgical Management

Surgery must be considered in endometriosis patients with persistent pain despite medical therapy or if there is any contraindication to its use [39]. Surgery is also necessary in cases of obstruction of the bowel or urinary tract due to endometriotic adhesions or in cases of exclusion of malignancy in an adnexal mass [40,41]. Surgical treatment (laparoscopy or robotic, preferably) determines a definitive diagnosis of endometriosis, and a conservative approach is preferred. However, the recurrence rate is relevant, estimated as 40 to 50% at 5 years after surgery [42].

Surgery may affect fertility because of the inherent alterations in vascularization and the direct manipulation of different structures, such as the ovaries, which can affect ovarian reserve [43,44].

These limitations on endometriosis treatment have stimulated growing interest in the biological mechanisms that sustain lesion survival, particularly metabolic reprogramming.

## 3. Energy Pathways: Glycolytic and Oxidative Fluxes

**Figure 2:**
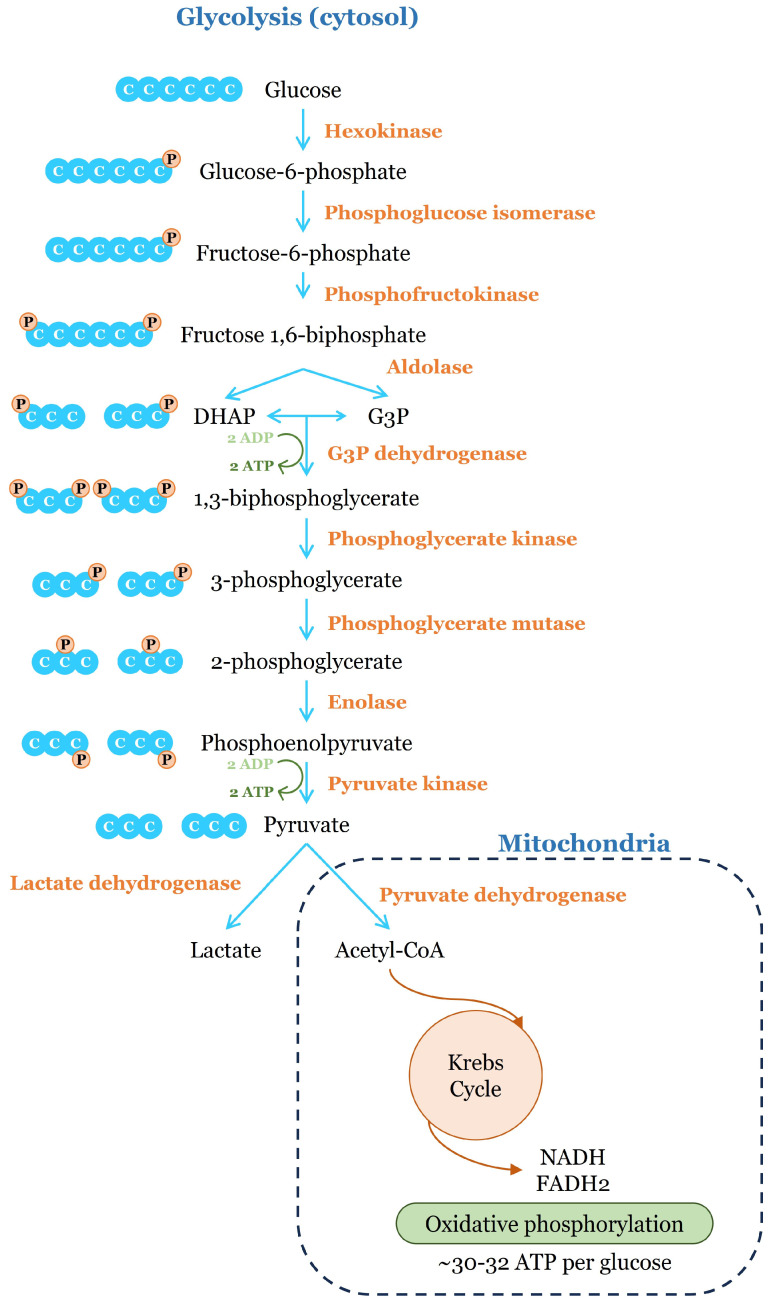
Simplified overview of the glycolytic pathway. Glucose enters the cell via glucose transporters (GLUTs) and undergoes a series of enzymatic reactions to produce pyruvate, ATP, and metabolic intermediates required for cellular growth and biosynthesis. Under aerobic conditions, pyruvate is primarily directed to mitochondrial oxidative phosphorylation, whereas under hypoxic conditions or during metabolic reprogramming, it may be preferentially converted to lactate. The figure highlights key enzymes and intermediates relevant to the discussion of glycolytic alterations in endometriosis. ATP: adenosine triphosphate; DHAP: dihydroxyacetone phosphate; FADH2: flavin adenine dinucleotide (reduced form); G3P: glyceraldehyde 3-phosphate; NADH: nicotinamide adenine dinucleotide (reduced form). Original figure created by the authors.

Cells use nutrients to generate energy-rich molecules, such as adenosine triphosphate (ATP), for essential functions, with glucose as the primary substrate. Glycolysis and respiration (Figure 2) represent the core pathways of glucose metabolism [45,46]. Glucose enters cells via glucose transporters (GLUTs) and is phosphorylated to glucose-6-phosphate by hexokinase (ubiquitous enzyme). This key metabolite then undergoes glycolysis—10 sequential steps divided into an energy-investment phase (steps 1–5: −2 ATP) and a payoff phase (steps 6–10: +4 ATP, +2 NADH net gain)—yielding pyruvate. Critical regulatory enzymes include hexokinase, phosphofructokinase-1 (rate-limiting), and pyruvate kinase [47].

Depending on the cellular microenvironment and oxygen availability, pyruvate may follow one of two different fates: it may be converted into lactate by lactate dehydrogenase A (LDHA), or it may enter the mitochondria for oxidative phosphorylation (OXPHOS), being decarboxylated to acetyl-CoA by the pyruvate dehydrogenase complex [48,49], fueling the Krebs cycle and subsequent electron transport chain [50]. This process is much more energy-efficient than glycolysis alone (yielding ~30–32 ATP per glucose via complete oxidation), but it requires oxygen consumption (an aerobic process) [47]. On the other hand, lactate must be exported for use by other cells, with its extrusion and uptake facilitated by monocarboxylate transporters (MCTs) [51].

MCTs belong to the Solute Carrier 16A (SLC16A) family of proton-linked plasma membrane transporters. There are 14 members identified so far, but MCT1 and MCT4 are the most studied lactate–proton symporters [52]. For transport to occur, MCTs first bind a hydrogen ion and then bind the monocarboxylate (lactate, ketone bodies, pyruvate, etc.) [53]. MCT1 and MCT4 exhibit different affinities for monocarboxylates: MCT1 has higher substrate affinity and is classically associated with both lactate uptake and efflux. MCT4 is a low-affinity transporter, usually overexpressed in cells with high glycolytic activity, mediating lactate efflux. Their activity and expression are dependent on the association with the chaperone protein CD147 (also known as basigin) [54].

## 4. Glucose Metabolism Reprogramming in Endometriosis

Recent studies have highlighted the potential role of altered glucose metabolism in the pathogenesis of endometriosis, presenting a novel opportunity to understand its underlying mechanisms and opening doors to new therapeutic targets [55,56]. This is thought to happen because of the hypoxic microenvironment and oxidative stress that can induce metabolic reprogramming in these cells [57].

### 4.1. Glycolysis Versus OXPHOS in Endometriotic Cells: The Warburg-like Effect

Originally proposed by Warburg as the cancer cell’s preference for glycolysis—converting glucose to lactate even in oxygen-rich conditions [58], a Warburg-like metabolic phenotype has recently been described in endometriosis as a metabolic shift from OXPHOS to glycolysis. Mechanistically, it can be driven by the TGF-β–HIF-1 system, a well-described cancer-related axis. Hypoxia-inducible factor-1 (HIF-1) and transforming growth factor-beta (TGF-β) increase LDHA expression, promoting lactate production from pyruvate and increasing pyruvate dehydrogenase kinase (PDK) activity, which in turn deactivates pyruvate dehydrogenase (PDH) [59], inhibiting acetyl-CoA production from pyruvate. Consequently, this leads to reduced mitochondrial energy production and reactive oxygen species (ROS) generation. Hence, the Warburg-like effect suppresses ROS overproduction, activates cell survival signals, and thus prevents endometriotic cell death [56,60].

**Figure 3:**
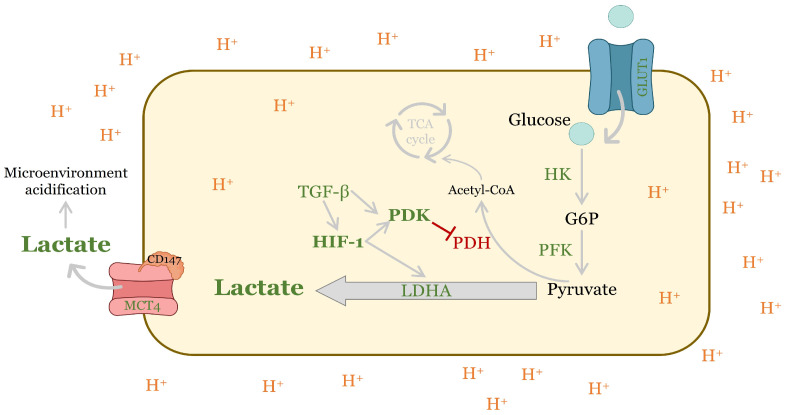
Proposed mechanisms of glycolytic reprogramming in endometriosis. Hypoxia, inflammatory mediators, and growth factor signaling promote a shift toward enhanced glycolytic metabolism in endometriotic lesions. Increased expression of glucose transporters, glycolytic enzymes, PDK, LDHA, and MCTs contributes to increased glucose utilization and lactate production. These metabolic alterations have been associated with enhanced cellular proliferation, angiogenesis, resistance to apoptosis, immune evasion, and lesion persistence. Green letters indicate increased levels of enzymes and metabolites; red letters indicate decreased enzyme activity. CD147, cluster of differentiation 147; G6P, glucose 6-phosphate; GLUT, glucose transporter; HIF-1, hypoxia-inducible factor-1; HK, hexokinase; LDHA, lactate dehydrogenase A; MCT, monocarboxylate transporter; PDH, pyruvate dehydrogenase; PDK, pyruvate dehydrogenase kinase; PFK, phosphofructokinase; TCA, tricarboxylic acid; TGF-β, Transforming Growth Factor-Beta. Original figure created by the authors using BioRender.

Several findings corroborate the previous hypothesis. Some studies have shown increased gene expression of PDK1, LDHA, and HIF-1 in endometriotic cells compared with the eutopic endometrium [56,60,61]. However, most available evidence derives from cell culture and experimental models. Lee et al. showed that hypoxia plays an important role in increasing HIF-1 levels, which in turn increase PDK1 expression (Figure 3) [57]. Moreover, as PDK1 increases, oxidative stress-induced apoptosis decreases, supporting the notion that the metabolic switch in endometriosis likely results from hypoxia, with these changes driven by the microenvironment [57,60,62]. Moreover, Kasvandik et al. showed that ectopic stromal cells (compared with eutopic stromal cells) exhibit a lower oxygen consumption rate and increased lactate production [60].

Overall, to summarize the importance of glucose metabolism in endometriosis, lactate overproduction and accumulation in the microenvironment [56,63], increased glucose consumption, and increased expression of glycolysis-related enzymes in endometriotic cells [64] should be highlighted.

As stated above, endometriosis is a heterogeneous disease comprising superficial peritoneal lesions, ovarian endometriomas, and deep infiltrating endometriosis. However, most studies investigating glycolytic reprogramming have focused on selected lesion types, particularly ovarian endometriomas and stromal cell cultures. Consequently, it remains unclear whether the reported metabolic alterations are uniformly present across all endometriosis phenotypes.

Fluorodeoxyglucose–positron emission tomography (^18^FDG-PET) imaging may hold potential as a non-invasive tool for detecting endometriosis by targeting metabolic activity or specific molecular markers in lesions. This technique detects radioactivity emitted after a small amount of a radioactive tracer is injected into the bloodstream and can also assess glucose consumption in different tissues [65]. There are currently no published studies evaluating the effectiveness of ^18^FDG-PET in assessing patients with endometriosis. However, in some studies, ^18^FDG-PET images of women with endometriosis have identified abdominopelvic endometriotic lesions [65,66]. Although preliminary findings are encouraging, the available studies are retrospective and involve cohorts with very small sample sizes, in which PET scans were performed for distinct reasons (rather than as part of an attempt to diagnose endometriosis). No standardized clinical application has yet been established.

### 4.2. Players of Glucose Metabolism in Endometriotic Cells—Available Evidence

As in physiological settings, glucose enters endometriotic cells via facilitated diffusion, with GLUTs mediating its uptake. GLUTs are encoded by the Solute Carrier Family 2 (SLC2) gene family and include 14 members, of which GLUT1-4 have high affinity for glucose [67]. One study showed that, like cancer cells, GLUT expression seems to be increased in endometriotic cells: GLUT3 and 4 are more expressed in ectopic lesions than in eutopic endometrium, and strong GLUT1 immunoreactivity was observed in ectopic lesions [67]. Current evidence remains limited by the small number of available studies and the heterogeneity of methodologies.

As previously mentioned, MCTs mediate lactate extrusion, avoiding intracellular acidification. MCT4 is the main lactate extruder in glycolytic cells, and although it plays a major role in glucose metabolism, it has been scarcely studied in endometriosis. In an experimental model of endometriosis, Bahrami et al. demonstrated the expression of GLUT1, GLUT3, MCT1, and MCT4 in ectopic endometrial tissue, suggesting a symbiosis between glucose and monocarboxylate transporters and neovascularization in endometriotic lesions [68]. Regarding MCTs’ chaperone basigin, there are conflicting results on its expression in endometriotic tissues. Besides maintaining MCTs at the cell-surface membrane, basigin induces the expression of angiogenic factors and the activity of matrix metalloproteinases and regulates tissue remodeling both in physiological and pathological settings, including endometriosis [69]. Braundmeier et al. and Smedts et al. observed that basigin expression was increased in ectopic endometrium compared to eutopic endometrium, hypothesizing that it may promote disease progression because it may facilitate its implantation, vascularization, and growth [70,71]. However, basigin increased expression may not be a predisposing factor for endometriosis, as its location is the same in the eutopic endometrium of women with and without endometriosis [71]. Nevertheless, a different quantitative analysis performed by Braundmeier et al. demonstrated that basigin expression was higher in the eutopic endometrium of women with endometriosis than in healthy women [72]. Regarding ovarian tissue, a marked basigin expression was shown in women with endometriosis [71].

## 5. Similarities Between Cancer and Endometriosis

**Figure 4:**
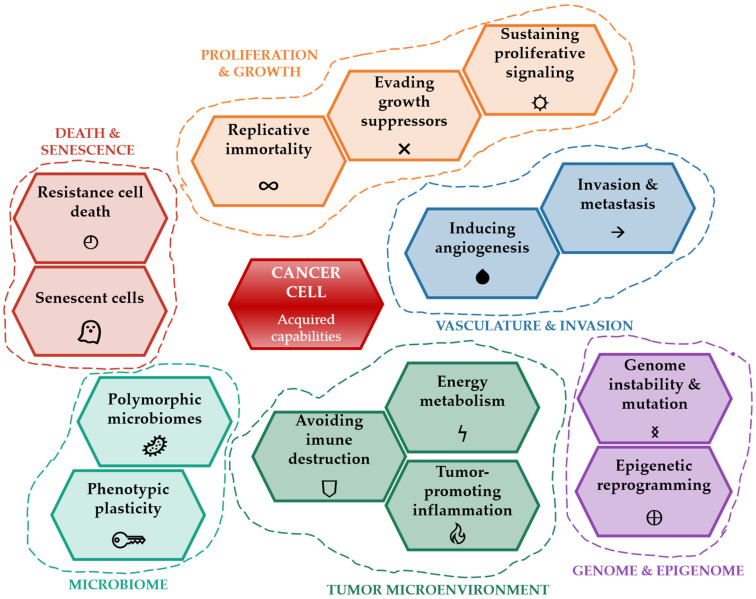
The hallmarks of cancer. Endometriotic lesions exhibit biological features that partially overlap with established hallmarks of cancer, including sustained proliferative signaling, resistance to apoptosis, angiogenesis, immune evasion, invasion of surrounding tissues, chronic inflammation, and metabolic reprogramming. However, these similarities represent shared cellular and molecular mechanisms rather than biological equivalence, as endometriosis remains a benign and highly heterogeneous disease with distinct clinical behavior. The figure summarizes some common features that may contribute to understanding disease persistence and to the identification of novel therapeutic targets. For clarity, the hallmarks are grouped according to the major biological processes they primarily influence. The colour-coded dashed outlines highlight these functional clusters and illustrate the close biological interconnection between related hallmarks. Orange, proliferation and growth; blue, vasculature and invasion; green, tumor microenvironment; purple, genome and epigenome; red, death and senescence; turquoise, microbiome. Original figure created by the authors.

The hallmarks of cancer, first outlined by Hanahan and Weinberg in 2000 [73] and updated later [74,75,76], describe core capabilities that enable malignant transformation and tumor progression (Figure 4) [76]. These traits allow cancer cells to evade normal controls, also reflecting deeper insights into immunity and metabolism. In fact, deregulating cellular metabolism is an established hallmark, where cancer cells reprogram their metabolism to prioritize biomass production and survival over efficient energy generation.

Although respiration is the predominant metabolic energy-producing pathway in normal cells, most cancer cells exhibit a glycolytic profile, presenting a high rate of glucose consumption even in the presence of normal oxygen levels, a phenomenon known as the Warburg effect since it was first proposed by Otto Warburg in the 1920s [58]. Increased glycolysis rates lead to increased lactate production by cancer cells. Impaired angiogenesis and oxygenation, as well as oncogenic activation, are responsible for this metabolic switch [77,78]. Therefore, more lactate molecules will be produced, resulting in acidification of the tumor microenvironment and promoting cell proliferation, migration, invasion, immune system modulation, and angiogenesis. Ultimately, cancer cell survival will increase [78].

Despite sharing several mechanistic features with cancer (Table 1), endometriosis remains a benign, estrogen-dependent, and highly heterogeneous disease with a fundamentally different biological behavior. Therefore, the comparison with cancer should be interpreted as a framework for understanding common cellular and molecular mechanisms rather than as evidence of biological equivalence. Regarding cell proliferation in cancer cells, oncogenes are responsible for self-sufficiency to grow, independently of diffusible growth factors, extracellular matrix components, and cell-to-cell adhesion/interaction molecules [76]. In endometriosis, there are also some (still unknown) stimuli that lead to excessive endometrial cell growth outside the uterine cavity. Both conditions are also associated with inflammation and immune dysfunction. Within the complex tumor microenvironment, immune cells will be altered, and their functions will be affected: macrophages and leukocytes chronically produce ROS, which are deleterious and perpetuate tissue damage and cellular proliferation. These cells may release tumor necrosis factor-alpha (TNF-α) and macrophage migration inhibitory factor (MIF) to exacerbate deoxyribonucleic acid (DNA) damage and impair p53-dependent protective responses. In endometriosis, among other immune changes, there is an imbalance between pro-inflammatory [interleukins (ILs) 1, 6, 8, and TNF-α] and anti-inflammatory (IL10 and TGF-β) cytokines and a switch from natural killer cells (NK) surveillance to NK suppression, which causes a decrease in cytotoxicity to endometrial cells [79,80]. Angiogenesis is also enhanced in both diseases, playing a pivotal role in disease progression by supplying ectopic lesions and tumors with essential nutrients and oxygen while facilitating the removal of metabolic waste. For instance, vascular endothelial growth factor (VEGF) A levels are increased in both cancer and endometriosis [74,81]. Regarding hormonal dependence, it is well known that in endometriosis there is an excess of estradiol, a well-recognized mitogenic factor, promoting cell proliferation and decreased apoptosis. In some cancers, such as endometrial, breast, and ovarian cancers, tumor growth is also dependent on this hormone. Infiltration and invasion are also common characteristics of both cancer and endometriosis: cancer cells invade surrounding tissues and can spread to other organs (lymphatic or hematogenous dissemination) [82,83]. In endometriosis, the term “metastasis” does not apply. Still, as mentioned above, there may be endometriotic cells distant from the pelvic region, confirming the presence of some lesion spread [17,18,19,20,21]. Invasion occurs in both diseases and causes adhesions and distortion of the normal anatomy, and because of them, different symptoms will appear, resulting from the infiltration and the inflammatory response induced in nearby structures [76,84].

Importantly, these mechanistic similarities should not obscure the fundamental biological differences between endometriosis and cancer. Unlike malignant tumors, endometriosis does not exhibit unrestricted cellular proliferation, widespread genomic instability, or progressive loss of tissue architecture leading to organ failure. Lesion growth remains strongly influenced by hormonal and microenvironmental factors, and disease progression does not generally result in the life-threatening systemic complications that characterize cancer. Although endometriosis imposes a considerable burden through chronic pain, infertility, and impaired quality of life, its clinical course, prognosis, and mortality differ profoundly from those of malignant disease.

## 6. Future Directions: Novel Glycometabolism-Related Therapeutics Ahead

As explained above, endometriosis is a very challenging disease because it seems to have a multifactorial origin. As its pathophysiology has yet to be determined, it is a challenge to provide women with effective answers regarding treatment. Currently, there are only drugs for symptomatic treatment, which is not enough for a chronic and therefore symptomatic disease. In this setting, mysteries remain: the response to hormonal and analgesic therapy is very different among women and has no apparent relation to the clinical condition presented by each of them [22]. For this reason, studies are investigating the repurposing of certain drugs already used for other pathologies, particularly glycolytic metabolism inhibitors (Table 2), which target the Warburg-like metabolic reprogramming observed in endometriotic lesions to potentially disrupt lesion survival and growth beyond symptomatic control.

A very significant obstacle associated with many of these drugs is the unfavorable toxicity profile due to their ubiquitous expression [85]. Despite the growing interest in glycolysis-targeted therapies, several challenges must be addressed before their clinical application in endometriosis. First, many of the proposed targets, including GLUTs, MCTs, LDHA, and PDK, are not disease-specific and are broadly expressed in normal tissues. Consequently, systemic inhibition may interfere with physiological metabolic processes and lead to off-target toxicity. Another important limitation is metabolic plasticity. Cells frequently compensate for glycolytic inhibition by activating alternative pathways, including glutaminolysis, fatty acid oxidation, and other biosynthetic networks. Such adaptive responses may reduce therapeutic efficacy and contribute to treatment resistance.

Furthermore, most currently available evidence derives from in vitro studies and animal models, while human data remain extremely limited. Disease heterogeneity represents an additional challenge, as it remains unclear whether metabolic alterations are equally relevant across superficial peritoneal lesions, ovarian endometriomas, and deep infiltrating endometriosis.

Finally, no validated biomarkers currently exist to identify patients who may benefit from metabolism-directed therapies or to predict treatment response. Future research should therefore focus not only on target validation and safety assessment but also on developing biomarkers to support patient selection and personalized therapeutic strategies.

Table 2 summarizes some information on the main possible targets.

### 6.1. MCT Inhibitors

Although direct evidence in endometriosis remains scarce, several observations suggest that MCTs may contribute to lesion establishment and persistence. Endometriotic lesions display increased glycolytic activity and lactate production, requiring efficient lactate export mechanisms. Moreover, basigin, the main chaperone required for MCT membrane localization and activity, has been reported to be overexpressed in ectopic endometrium and ovarian endometriosis, supporting a potential role for MCT-mediated metabolic adaptation. A reduction in mRNA levels of MCT1/4 was observed following treatment with atorvastatin and resveratrol [86].

However, the ubiquitous expression of MCTs in normal tissues, particularly skeletal muscle, myocardium, and erythrocytes, raises important concerns regarding systemic toxicity and therapeutic selectivity.

### 6.2. GLUT-Targeted Approaches

Given the increased glucose demand associated with glycolytic reprogramming, glucose transporters have emerged as potential therapeutic targets in endometriosis.

Atorvastatin and resveratrol have attracted interest as potential modulators of glycolytic metabolism in endometriosis. In experimental models, both compounds reduced GLUT1 and GLUT3 expression and decreased angiogenesis and lesion development. Similarly, Gui-Zhi-Fu-Ling capsules (a traditional Chinese herbal formulation) were associated with reduced GLUT4 expression [68,86].

### 6.3. LDHA and PDK Inhibition: Targeting the Glycolytic Phenotype

Given its central role in glycolysis, LDHA has emerged as an attractive therapeutic target in oncology, with several experimental inhibitors that reduce lactate production, cellular proliferation, and tumor growth in preclinical models. However, despite evidence suggesting increased LDHA activity in endometriosis, specific studies evaluating LDHA inhibitors in endometriotic lesions remain unavailable. Therefore, the potential efficacy and safety of direct LDHA inhibition in endometriosis remain largely speculative and warrant further investigation.

PDK represents another promising metabolic target.

Among the available PDK inhibitors, dichloroacetate (DCA) has attracted particular interest. DCA promotes PDH activation by inhibiting PDK activity, thereby redirecting pyruvate metabolism towards mitochondrial oxidative phosphorylation and reducing lactate production. In endometriosis models, DCA has been shown to decrease lactate secretion, reduce peritoneal fluid lactate concentrations, inhibit cellular proliferation, and reduce lesion size [87]. These findings provide some of the strongest preclinical evidence supporting metabolism-based therapeutic strategies in endometriosis. These promising findings led to the EPiC1 clinical trial, a single-arm feasibility study evaluating DCA for endometriosis-associated pain in women, with the randomized placebo-controlled EPiC2 trial currently ongoing [88].

Nevertheless, several challenges remain before PDK inhibition can be translated into clinical practice. DCA has been investigated in multiple non-oncological and oncological settings, and although generally well tolerated, prolonged exposure has been associated with peripheral neuropathy, gastrointestinal symptoms, and occasional hepatotoxicity. Furthermore, the long-term consequences of manipulating mitochondrial metabolism in women with endometriosis remain unknown.

## 7. Conclusions

Current evidence supports glycolytic alterations in endometriosis and suggests that metabolic reprogramming may contribute to lesion persistence and disease progression. However, most available data remain preclinical, and important questions regarding disease heterogeneity, target validation, safety, and clinical applicability remain unresolved.

Further translational and clinical studies must focus on identifying and investigating the pathways underlying endometriosis pathogenesis, which may enable the long-term use of drugs targeting specific pathways rather than merely providing symptomatic treatment. The striking parallels with cancer—including evidence of dysregulated glucose metabolism—open avenues for repurposing glycolytic inhibitors, successfully tested in malignant settings, to disrupt survival and progression of endometriotic lesions.

## Figures and Tables

**Table 1:** Comparative overview of the shared biological features of cancer and endometriosis.

Feature	Cancer	Endometriosis
Cell proliferation	Oncogene-driven self-sufficient growth	Excessive endometrial cell growth outside the uterine cavity
Metabolic reprogramming	Warburg effect: high glycolysis and lactate production even under normoxia	Glycolytic profile with lactate overproduction and increased glycolysis-related enzymes
Inflammation and immune dysfunction	Chronic ROS production by macrophages/leukocytes; TNF-α and MIF release; DNA damage	Pro-/anti-inflammatory cytokine imbalance; NK cell suppression with decreased cytotoxicity
Angiogenesis	Increased VEGF-A; neovascularization supplying tumors	Increased VEGF-A; neovascularization supplying ectopic lesions
Hormonal dependence	Estradiol-dependent growth (endometrial, breast, ovarian cancers)	Estradiol excess promoting proliferation and decreased apoptosis
Invasion and dissemination	Tissue invasion with lymphatic/hematogenous metastasis	Infiltration with ectopic implants in distant sites (without true metastasis)
Adhesions and anatomical distortion	Invasion causes adhesions and anatomical distortion	Adhesions and anatomical distortion from infiltration and inflammation

**Table 2:** Summary of the main targets, their proposed role in endometriosis, available evidence, observed effects, limitations, and potential translational relevance.

Target	Mechanism	Evidence	Observed Effects	Limitations and Toxicity Concerns	Translational Potential
MCT1/ MCT4	Lactate export and maintenance of glycolytic phenotype	Experimental mechanistic	Microenvironment modulation: potential regulation of extracellular acidification, angiogenesis and lesion survival	Inhibition may impair exercise tolerance and cardiac metabolism	Moderate
GLUT1/ GLUT3	Increased glucose uptake supporting enhanced glycolytic activity	Preclinical	Increased expression in ectopic lesions; reduction after atorvastatin and resveratrol treatment	Fatigue, neuroglycopenic symptoms and metabolic disturbances	Moderate
PDK	Inhibition of pyruvate dehydrogenase	Preclinical	Increased expression in endometriotic cells; dichloroacetate reduced lactate secretion, lesion size and cell proliferation	Peripheral neuropathy, gastrointestinal toxicity and hepatotoxicity (prolonged exposure)	High
LDHA	Conversion of pyruvate into lactate and maintenance of aerobic glycolysis	Experimental mechanistic	Increased expression in ectopic stromal cells and association with lactate overproduction. Inhibition: reduced glycolytic flux	Inhibition may impair skeletal muscle adaptation and cellular energy homeostasis	Moderate
HIF-1	Upstream regulator of glycolytic reprogramming under hypoxic conditions	Preclinical	Reduced glycolytic switch	Pleiotropic effects: broad physiological functions in angiogenesis and cellular adaptation to hypoxia raise concerns regarding off-target effects	Low
TGF-β	Upstream mediator of Warburg-like metabolic reprogramming	Experimental mechanistic	Promotion of glycolytic switch and lactate production	Pleiotropic role in immune regulation and tissue homeostasis may limit therapeutic targeting	Low

## Data Availability

No new data were created or analyzed in this study. Data sharing is not applicable to this article.

## Abbreviations

The following abbreviations are used in this manuscript:

AI	Aromatase Inhibitors
ATP	Adenosine Triphosphate
COX	Cyclooxygenase
CD147	Cluster of differentiation 147
DCA	Dichloroacetate
DHAP	Dihydroxyacetone Phosphate
DNA	Deoxyribonucleic Acid
FADH2	Flavin Adenine Dinucleotide
^18^FDG-PET	Fluorodeoxyglucose–Positron Emission Tomography
G3P	Glyceraldehyde 3-Phosphate
G6P	Glucose 6-Phosphate
GLUT	Glucose Transporter
GnRH	Gonadotropin-Releasing Hormone
HIF-1	Hypoxia-Inducible Factor-1
HK	Hexokinase
IL	Interleukin
LDHA	Lactate Dehydrogenase A
MCT	Monocarboxylate Transporter
MIF	Macrophage migration Inhibitory Factor
NADH	Nicotinamide Adenine Dinucleotide
NK	Natural Killer Cells
NSAIDs	Nonsteroidal Anti-Inflammatory Drugs
OXPHOS	Oxidative Phosphorylation
PDH	Pyruvate Dehydrogenase
PDK	Pyruvate Dehydrogenase kinase
PET	Positron Emission Tomography
PFK	Phosphofructokinase
ROS	Reactive Oxygen Species
SLC16A	Solute Carrier 16A
SLC2	Solute Carrier Family 2
TCA	Tricarboxylic Acid
TGF-β	Transforming Growth Factor-Beta
TNF-α	Tumor Necrosis Factor-Alpha
VEGF	Vascular Endothelial Growth Factor
